# 3D Electron Microscopy Study of Synaptic Organization of the Normal Human Transentorhinal Cortex and Its Possible Alterations in Alzheimer’s Disease

**DOI:** 10.1523/ENEURO.0140-19.2019

**Published:** 2019-07-09

**Authors:** M. Domínguez-Álvaro, M. Montero-Crespo, L. Blazquez-Llorca, J. DeFelipe, L. Alonso-Nanclares

**Affiliations:** 1 Laboratorio Cajal de Circuitos Corticales, Centro de Tecnología Biomédica, Universidad Politécnica de Madrid, Madrid 28223, Spain; 2 Instituto Cajal, Consejo Superior de Investigaciones Científicas (CSIC), Madrid 28002, Spain; 3 Departamento de Psicobiología, Facultad de Psicología, Universidad Nacional de Educación a Distancia (UNED), Madrid 28040, Spain; 4 Centro de Investigación Biomédica en Red sobre Enfermedades Neurodegenerativas (CIBERNED), Madrid 28031, Spain

**Keywords:** dementia, dendritic shafts, dendritic spines, FIB/SEM, synaptic morphology

## Abstract

The transentorhinal cortex (TEC) is an obliquely oriented cortex located in the medial temporal lobe and, together with the entorhinal cortex, is one of the first affected areas in Alzheimer’s disease (AD). One of the most widely accepted hypotheses is that synaptopathy (synaptic alterations and loss) represents the major structural correlate of the cognitive decline observed in AD. However, very few electron microscope (EM) studies are available; the most common method to estimate synaptic density indirectly is by counting, at the light microscopic level, immunoreactive puncta using synaptic markers. To investigate synaptic morphology and possible alterations related to AD, a detailed three-dimensional (3D) ultrastructural analysis using focused ion beam/scanning EM (FIB/SEM) was performed in the neuropil of Layer II of the TEC in human brain samples from non-demented subjects and AD patients. Evaluation of the proportion and shape of asymmetric synapses (AS) and symmetric synapses (SS) targeting spines or dendritic shafts was performed using 3D reconstructions of every synapse. The 3D analysis of 4722 synapses revealed that the preferable targets were spine heads for AS and dendritic shafts for SS, both in control and AD cases. However, in AD patients, we observed a reduction in the percentage of synapses targeting spine heads. Regarding the shape of synapses, in both control cases and AD samples, the vast majority of synapses had a macular shape, followed by perforated or horseshoe-shaped synapses, with fragmented synapses being the least frequent type. Moreover, comparisons showed an increased number of fragmented AS in AD patients.

## Significance Statement

Determination of postsynaptic targets, shape and size of the synaptic junctions provide critical data on synaptic functionality. However, as far as we know, detailed three-dimensional (3D) synaptic morphology and identification of postsynaptic targets in serial sections have not been performed before in the human brain. The present study represents the first attempt to unveil the synaptic organization of the neuropil of the human brain at the ultrastructural level using 3D electron microscopy (EM). Our present results provide a new, large, quantitative ultrastructure dataset of the synaptic organization of the normal human cortex and of the synaptic alterations that occur in Alzheimer’s disease (AD). Thus, these results may help to understand the relationship between alterations of the synaptic circuits and the cognitive deterioration in AD.

## Introduction

The transentorhinal cortex (TEC) is an obliquely oriented cortex located in the medial temporal lobe between the perirhinal cortex and the entorhinal cortex and is considered as a transitional zone between the periallocortex and the proisocortex (Braak and Braak, 1985; Braak et al., 1996; Insausti et al., 2017). This cortical region, together with the entorhinal cortex, is one of the first affected regions in Alzheimer’s disease (AD). AD is characterized by two hallmark lesions: extracellular amyloid-β (Aβ)-plaques and intracellular neurofibrillary tangles of filamentous aggregates of hyperphosphorylated tau protein (Alzheimer’s Association, 2018). In particular, neurofibrillary tangles first affect the TEC (Braak and Braak, 1991).

Aβ peptides and tau proteins play normal roles at the synapse, but under pathologic conditions, they may lead to toxic effects at both presynaptic and postsynaptic elements, leading to synaptic loss and causing dysfunction in neurotransmitter release (Spires-Jones and Hyman, 2014; Dorostkar et al., 2015; Henstridge et al., 2016; Rajmohan and Reddy, 2017; Zhou et al., 2017). At present, one of the most widely accepted hypothesis is that synaptopathy (synaptic alterations and loss) represents the major structural correlate of the cognitive decline observed in AD (Dickson et al., 1995; Sze et al., 1997; Masliah et al., 2001; Selkoe, 2002; Coleman et al., 2004; Arendt, 2009).

In a previous study, Domínguez-Álvaro et al. (2018) focused ion beam/scanning electron microscopy (FIB/SEM) was used to study the possible alterations of synapses in the neuropil of Layer II of TEC in human brain samples from AD patients. Unexpectedly, it was observed that there were no significant differences in the density, size and spatial distribution of synapses between AD and control samples. However, cortical thickness of the TEC in AD patients was reduced (35% thinner than in control subjects). Thus, a decrease in the total number of synapses in AD occurs in Layer II of the TEC. It was proposed that the surviving TEC neurons might display a compensatory mechanism (such as the generation of new dendritic branches), which would explain the lack of changes in the synaptic density.

It is well established that synapses are continuously formed, eliminated and/or reshaped in response to synaptic activity (Fauth and Tetzlaff, 2016). Synapses are dynamic elements that can change in terms of not only their function, but also their morphology and molecular features (Wegner et al., 2018). These synaptic morphological changes may include an increase in the number of perforated synapses [with one or more holes in the postsynaptic density (PSD)] or fragmented synapses (with several PSDs; Geinisman et al., 1987, 1991, 1992a,b). How these morphological changes affect the synaptic function is unknown, but since larger PSDs contain more receptors, it may be that these changes are related to changes in the synaptic transmission efficiency, as proposed for long-term potentiation (LTP; Lüscher et al., 2000).

Changes in the postsynaptic elements have also been described in AD patients and in animal models of AD, specifically, morphological alterations of dendritic spines (for simplicity, spines; Spires-Jones et al., 2007; Knafo et al., 2009; Tackenberg et al., 2009; Merino-Serrais et al., 2011, 2013; Pozueta et al., 2013), which are the major targets of excitatory synapses in the cerebral cortex (for review, see DeFelipe, 2015). Whether or not synaptic loss precedes neuronal loss by synaptic disconnection is not known, but previous studies on the morphological alterations in spines of pyramidal cells in AD patients (Merino-Serrais et al., 2013) appear to support the notion of synaptic loss preceding neuronal loss.

Finally, as far as we know, detailed three-dimensional (3D) synaptic morphology and identification of postsynaptic targets in serial sections have not been performed before in the human brain in either control cases or in AD patients. Thus, in the present study, FIB/SEM was used to determine the proportion and shape of asymmetric synapses (AS) and symmetric synapses (SS) targeting spines or dendritic shafts of 4722 synapses that were 3D reconstructed from the neuropil of Layer II of the TEC, from AD patients and control (non-demented) subjects. Accordingly, the present study represents the first attempt to unveil the synaptic organization of the neuropil of the human brain in both control and AD. This study provides a new, large, quantitative ultrastructure dataset of the synaptic organization of the normal human cortex and of the possible synaptic alterations that occur in AD.

## Materials and Methods

### Tissue preparation

Human brain tissue was obtained at autopsy from three sources: Pathologic Anatomy Service of Bellvitge University Hospital (Barcelona, Spain); Centro Alzheimer Fundación Reina Sofía, CIEN Foundation (Madrid, Spain); and from the Unidad Asociada Neuromax, Laboratorio de Neuroanatomía Humana, Facultad de Medicina, Universidad de Castilla-La Mancha, Albacete and the Laboratorio Cajal de Circuitos Corticales UPM-CSIC (Madrid, Spain). The tissue was obtained following national laws and international ethical and technical guidelines on the use of human samples for biomedical research purposes. Briefly, tissue samples were obtained from five control cases (with no recorded neurological or psychiatric alterations) and five AD patients according to the neuropathological criteria provided by the above-mentioned centers. As outlined in a previous study, immunostaining for anti-PHF-_Tau_ and anti-Aβ in the same control cases revealed no presence of Aβ-plaques and only occasional PHF-_Tau_ neurons. By contrast, in AD patients, the same immunostaining revealed the presence of a variable number of immunoreactive PHF-_Tau_ neurons and Aβ-plaques (Domínguez-Álvaro et al., 2018, their Table 1).

**Table 1.** Clinical and neuropathological information

Patient	Gender	Age (years)	Cause of death	Postmortem delay (h)	Braak stage	CERAD stage	Neuropsychological diagnosis
AB1	Male	45	Lung cancer	<1	NA	NA	NA
AB2	Female	53	Pulmonary shock	4	NA	NA	NA
IF10	Male	66	Bronchopneumonia plus cardiac failure	2	NA	NA	NA
M16	Male	40	Traffic accident	3	NA	NA	NA
M17	Male	36	Bronchopneumonia	2.5	NA	NA	NA
IF1	Female	80	–	2	IV	B	No evidence of cognitive impairment and dementia
IF2	Female	94	Pulmonary tuberculosis	1.5	V	C	Dementia
IF6	Male	85	Pneumonia	2	III	A	Mild cognitive impairment
VK11	Female	87	Respiratory inflammation	1.5	III−IV	A	Dementia
VK22	Female	86	–	2	V	C	Dementia

Braak Stages (Braak and Braak, 1991): III (NFTs in entorhinal cortex and closely related areas); III-IV (NFTs abundant in amygdala and hippocampus. Extending slightly into association cortex); V-VI (NFTs widely distributed throughout the neocortex and ultimately involving primary motor and sensory areas). CERAD Stages (Mirra et al., 1991): A (Low density of neuritic plaques); B (Intermediate density of neuritic plaques); C (High density of neuritic plaques). NA: Not applicable; NFTs: neurofibrillary tangles. – : Not available.

In all cases, the time between death and tissue processing was lower than 4h (Table 1). On removal, brain tissue was fixed in cold 4% paraformaldehyde (Sigma-Aldrich) in 0.1 M sodium phosphate buffer (PB; Panreac, 131965), pH 7.4, for 24-48 h. After fixation, the tissue was washed in PB and coronally sectioned in a vibratome (150-μm thickness; Vibratome Sectioning System, VT1200S Vibratome, Leica Biosystems). Sections containing TEC were selected and processed for EM as described elsewhere (Domínguez-Álvaro et al., 2018). Briefly, fixed sections were treated with 1% OsO_4_ (Sigma, O5500), 0.1% potassium ferrocyanide (Probus, 23345) and 0.003% CaCl_2_ in sodium cacodylate buffer (0.1 M) for 1 h at room temperature. After washing in PB, sections were stained with 2% uranyl acetate (EMS, 8473), and then dehydrated and flat-embedded in Araldite (TAAB, E021; DeFelipe and Fairén, 1993). Embedded sections were glued onto a blank Araldite block and trimmed. Semithin sections (1–2 μm thick) were obtained from the surface of the block and stained with 1% toluidine blue (Merck, 115930) in 1% sodium borate (Panreac, 141644). The last semithin section (which corresponds to the section immediately adjacent to the block surface) was examined under light microscope and photographed to accurately locate the neuropil regions to be examined. Tissue shrinkage due to EM processing was estimated by measuring the area before and after processing to correct the final values in both control and AD cases (Merchán-Pérez et al., 2009). The area after processing was divided by the area value measured before processing to obtain a shrinkage factor for any area measurement (p^2^) of 0.933.

### 3D EM

3D samples of Layer II from the TEC were obtained from all cases using a dual beam microscope (Crossbeam Neon40 EsB, Carl Zeiss NTS GmbH). This instrument combines a high-resolution field-emission SEM column with a focused gallium ion beam (FIB), which permits removal of thin layers of material from the sample surface on a nanometer scale. As soon as one layer of material is removed by the FIB (20 nm thick), the exposed surface of the sample is imaged by the SEM, using the backscattered electron detector. The sequential automated use of FIB milling and SEM imaging allowed us to obtain long series of photographs of a 3D sample of selected regions (Merchán-Pérez et al., 2009). Image resolution in the *xy* plane was 5 nm/pixel. Resolution in the z-axis (section thickness) was 20 nm, and image size was 2048 × 1536 pixels. The number of sections per stack ranged from 149 to 472, which corresponds to a corrected volume ranging from 260.2 to 824.4 μm^3^ (mean: 471.3 μm^3^). A total of 30 stacks of images of the neuropil from Layer II of the TEC were obtained (three stacks per case, for all 10 cases; total volume studied: 14,140 μm^3^). These images from the neuropil were obtained avoiding the neuronal and glial somata, blood vessels and, in the case of AD cases, also avoiding Aβ-plaques (plaque-free regions) to avoid the effect of alterations of synapses in the vicinity of Aβ-plaques, which has been described previously (Blazquez-Llorca et al., 2013).

### Synaptic 3D analysis

Stacks of images obtained by the FIB/SEM were analyzed using EspINA software (EspINA Interactive Neuron Analyzer, 2.1.9; http://cajalbbp.cesvima.upm.es/espina/); 3D reconstructed synapses were classified as AS (excitatory) or SS (inhibitory) based on their prominent or thin PSD, respectively (Gray, 1959; Colonnier, 1968; Peters and Palay, 1996).

EspINA software calculated the size of every synapse and also extracted the synaptic apposition surface (SAS) area, which provides the morphological features of the synapse, as an indicative measurement of the synaptic functionality (Morales et al., 2013).

**Figure 1.**
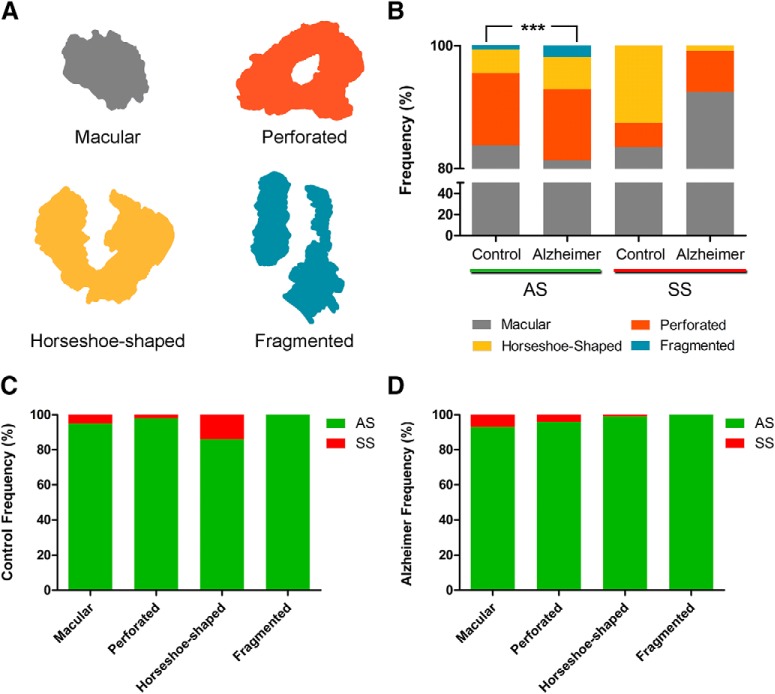
***A***, Schematic representation of the shape of the synaptic junctions: macular synapses with continuous disk-shaped PSD; perforated synapses with holes in the PSD; horseshoe-shaped with tortuous horseshoe-shaped perimeter with an indentation; and fragmented synapses with two PSDs with no connections between them. ***B***, Proportion of macular, perforated, horseshoe-shaped, and segmented AS and SS in control cases and AD patients. In AD patients, fragmented synapses were significantly more frequent than in control cases (χ^2^, *p* < 0.001). ***C***, Proportion of AS and SS belonging to each morphological category in control cases. The horseshoe-shaped synapses were significantly more frequent among SS than AS (χ^2^, *p* < 0.0001). ***D***, Proportion of AS and SS belonging to each morphological category in AD cases. *** *p* < 0.001.

EspINA was also used to visualize each of the reconstructed synapses in 3D and to detect the possible presence of perforations or deep indentations in their perimeters (an additional movie file shows this in more detail; Movie 1). Regarding the shape of the PSD, the synaptic junctions could be classified into four main types, according to Santuy et al. (2018a): macular (disk-shaped PSD); perforated (with one or more holes in the PSD); horseshoe-shaped (perimeter with an indentation) and fragmented (irregular small disk-shaped PSDs with no connection between them; Fig. 1*A*).

Movie 1. Video of the EspINA software user interface. FIB/SEM sections are viewed through the *xy*-plane (as obtained by FIB/SEM microscopy) and *yz*- and *xz*-plane. 3D reconstruction of a perforated synapse is shown in the three orthogonal planes. This 3D reconstruction of one perforated synapse is shown in green at the end of the video. The 3D reconstruction of the synapse allows us to identify the morphology of the synapse as perforated. 10.1523/ENEURO.0140-19.2019.movie.1

**Figure 2.**
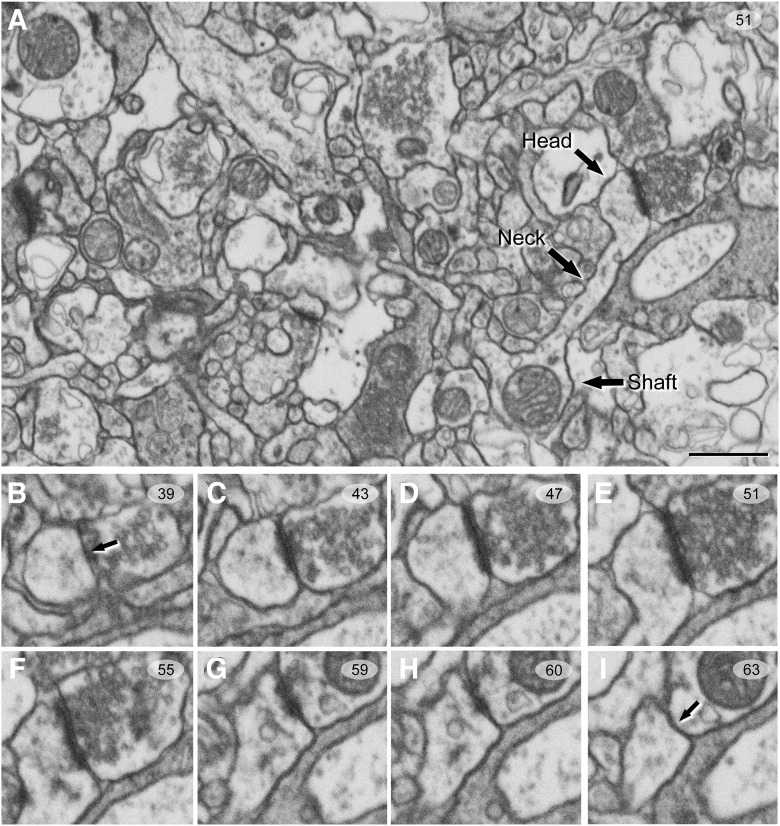
Serial images obtained by FIB/SEM from the human neuropil of Layer II of TEC. ***A***, Low-magnification photograph showing a spine head, spine neck, and dendritic shaft in a single image. ***B****−****I***, Selected sections (39–63) from an FIB/SEM stack of serial sections, to illustrate an AS targeting a spine head. Arrows (in ***B***, ***I***) indicate the beginning and the ending, respectively, of the synapse targeting a spine head. Scale bar, show in ***A***, indicates 850 nm in ***A*** and 500 nm in ***B−I***.

To identify the postsynaptic targets of 3D reconstructed synapses, the image stack was navigated to determine whether the postsynaptic element was a dendritic spine or a dendritic shaft. A condition for unambiguous identification of spines, versus dendritic shafts, was that the dendritic spine could be visually traced to the parent dendrite (Fig. 2). Accordingly, when the postsynaptic element of a synapse was close to the margins and it was truncated by the borders of the stack, the identity of the postsynaptic target could not be determined. Truncated elements that could not be conclusively identified were labeled as “unknown.” Therefore, the targets of synapses were classified into two main categories: dendritic spines and dendritic shafts. When the postsynaptic target was a dendritic spine, we further recorded the position of the synapse on the head or neck. We also determined whether the target dendrite was a spiny dendrite or a non-spiny one (Santuy et al., 2018b).

**Figure 3.**
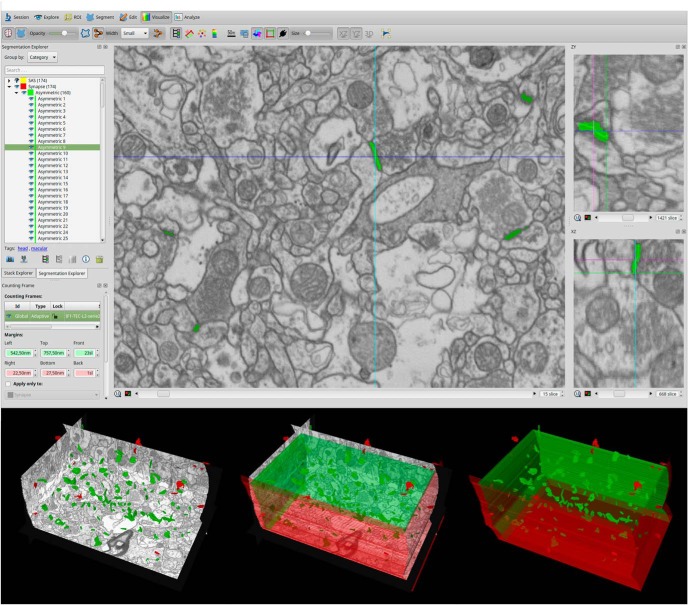
Screenshot of the EspINA software user interface. In the main window (top), the sections are viewed through the *xy*-plane (as obtained by FIB/SEM microscopy). The other two orthogonal planes, *yz* and *xz*, are also shown in adjacent windows (on the right). The 3D windows (bottom left) show the three orthogonal planes and the 3D reconstruction of segmented synapses. A 3D rectangular unbiased CF is shown on the bottom. The three acceptance planes are represented in green and three exclusion planes in red. Synapses inside the CF are colored in green and synapses outside the CF in red.

In addition, EspINA allowed the application of an unbiased 3D counting frame (CF) to perform direct counting (for details, see Merchán-Pérez et al., 2009). This 3D unbiased CF is a regular rectangular prism enclosed by three acceptance planes and three exclusion planes marking its boundaries (Fig. 3). All objects within the CF are counted, as are those intersecting any of the acceptance planes, while objects that are outside the CF, or intersecting any of the exclusion planes, are not counted. In this study, we have determined the morphology and postsynaptic target of 4722 synapses inside the 3D CF of the neuropil of Layer II of the TEC, from AD patients and control (with no recorded neurological or psychiatric alterations) subjects.

### Statistical analysis

To perform statistical comparisons of AS and SS proportions, χ^2^ test was used for contingency tables. The same method was used to study whether there were significant differences between groups in relation to the shape of the synaptic junctions and their postsynaptic target. Briefly, in all the χ^2^ statistical analyses, we firstly performed an “omnibus test” based on 2 × 4 contingency tables. To further investigate the specific cells driving the significance of the χ^2^ test, partitioning procedure was applied to create 2 × 2 contingency tables (Sharpe, 2015). To identify possible differences within a group regarding the synaptic size (SAS area, perimeter and ratio) related to the shape of the synapses and their postsynaptic target, a Kruskal–Wallis (KW) nonparametric test was performed (the normality and homoscedasticity criteria were not met). Differences in the synaptic size between groups were evaluated by using the unpaired Mann–Whitney (MW) nonparametric *U* test. Frequency distribution analysis of the SAS was performed using Kolmogorov–Smirnov (KS) nonparametric test. Statistical studies were performed with the aid of the GraphPad Prism statistical package (Prism 5.00 for Windows, GraphPad Software Inc.) and SPSS program (IBM SPSS Statistics v24, IBM Corp.).

## Results

Stacks of images obtained by the FIB/SEM were analyzed using EspINA software, which allows the segmentation of synapses in the reconstructed 3D volume (Morales et al., 2011). These TEC samples have been previously used to determine the density and size of synapses, as well as their spatial distribution (Domínguez-Álvaro et al., 2018). Since the synaptic junctions were fully 3D reconstructed, as described elsewhere (Merchán-Pérez et al., 2009), each synapse could be classified as AS (excitatory) or SS (inhibitory), based on its prominent or thin PSD, respectively (Gray, 1959; Colonnier, 1968; Peters et al., 1991; Peters and Palay, 1996). In these stacks of images, it was also feasible to accurately determine the synaptic targets, which is critical from the point of view of the synaptic organization.

### Distribution of postsynaptic targets

Postsynaptic targets were classified into two main categories: dendritic spines and dendritic shafts. In addition, when the postsynaptic target was a dendritic spine, we recorded the position of the synapse on the head or on the neck. When the postsynaptic target was a dendritic shaft, we also classified the dendrites as spiny or non-spiny, based on the presence or absence of spines, respectively.

#### Control

We determined the postsynaptic structures of 1396 AS whose target could be unambiguously identified as spines or dendritic shafts. Similarly, we analyzed a total of 112 SS.

In a previous study, it was found that AS outnumber SS ∼95:5 (Domínguez-Álvaro et al., 2018). In the present study, we observed that 59.1% of AS were established on spine heads and 0.5% on spine necks. The remaining AS were established on dendritic shafts: 40.4% (19.9% on aspiny dendritic shafts and 20.5% on spiny shafts; Table 2; Extended Data Table 2-1). SS showed a clearly different preference for postsynaptic targets: 92% were established on dendritic shafts (53.6% on spiny dendritic shafts and 38.4% on aspiny shafts). That is, the majority of SS target dendritic shafts, and a small percentage of SS were established on spine heads (7.1%) and spine necks (0.9%; Table 2; Extended Data Table 2-1).

**Table 2.** Distribution of AS and SS on spines and dendritic shafts in control cases and AD patients

Group	Type of synapse	Synapses on spine heads	Synapses on spine necks	Synapses on aspiny dendritic shaft	Synapses on spiny dendritic shaft	Total synapses
Control	AS	59.1% (825)	0.5% (7)	19.9% (278)	20.5% (286)	100% (1396)
	SS	7.1% (8)	0.9% (1)	38.4% (43)	53.6% (60)	100% (112)
Alzheimer	AS	50.2% (579)	0.7% (8)	28.0% (323)	21.1% (243)	100% (1153)
	SS	8.8% (9)	1.0% (1)	41.2% (42)	49.0% (50)	100% (102)

Synapses on spines have been sub-divided into those that are established on spine heads and those that are established on spine necks. Moreover, we differentiated between aspiny and spiny dendritic shafts. Data are expressed as percentages with the absolute number of synapses studied given in parentheses. Data for each individual case are represented in Table 2-1. Data expressed as absolute number of synapses were taken from this table to perform contingency tables showed in Table 2-2 and Table 2-3.

10.1523/ENEURO.0140-19.2019.t2-1 Extended Data Table 2-1 Distribution of AS and SS on spines and dendritic shafts for each individual case Download Table 2-1, DOCX file.

10.1523/ENEURO.0140-19.2019.t2-2 Extended Data Table 2-2 An example of a 2 × 4 contingency table showing the type of synapse against the type of postsynaptic target in control cases Download Table 2-2, DOCX file.

10.1523/ENEURO.0140-19.2019.t2-3 Extended Data Table 2-3 Three examples of 2 × 2 contingency tables showing the type of synapse against the type of postsynaptic target in control cases Download Table 2-3, DOCX file.

Therefore, we evaluated whether AS and SS had a preference for spines (heads and necks) or dendritic shafts. To evaluate this possibility, a 2 × 4 contingency table was created showing both types of synapses against the type of their postsynaptic target (Extended Data Table 2-2). The null hypothesis of this χ^2^ test was “H_0_: type of synapse and type of postsynaptic target are independent”; χ^2^ test demonstrated that the null hypothesis must be rejected (Pearson-χ^2^ = 117.06; df = 3; *p* < 0.0001), showing that there is an association between the type of synapses and the type of postsynaptic target. To determine which values in this test are driving this significance, 2 × 2 contingency tables were created (Extended Data Table 2-3). Since the observed values for SS in spine necks were <5, these data were discarded (Bewick et al., 2004). In the 2 × 2 tables, the expected counts of AS and SS on spine heads, aspiny dendritic shafts and spiny dendritic shafts were calculated from the marginal totals. In general, for any contingency table, the expected frequency for a cell in the *i*th row and the *j*th column is E*_ij_* = T*_i_*T*_j_*/T where T*_i_* is the marginal total for the *i*th row, T*_j_* is the marginal total for the *j*th column, and T is the total number of observations. χ^2^ tests were applied to these tables and the null hypothesis (“H_0_: type of synapse and type of postsynaptic target are independent”) was rejected; that is, there is an association between the type of synapse and the type of postsynaptic target. We found that AS had a significant preference for spine heads: 99.0% of AS and only 1.0% of SS were established on spine heads (χ^2^, *p* < 0.0001). By contrast, the SS showed a significant preference for dendritic shafts (χ^2^, *p* < 0.0001), both in aspiny dendritic shafts (receiving 86.6% AS and 13.4% SS) and spiny dendritic shafts (receiving 82.7% AS and 17.3% SS). Since the overall proportion of synapses on spines versus synapses on shafts was ∼56:44, each synaptic type, AS and SS, had a clear preference for a particular postsynaptic target.

**Figure 4.**
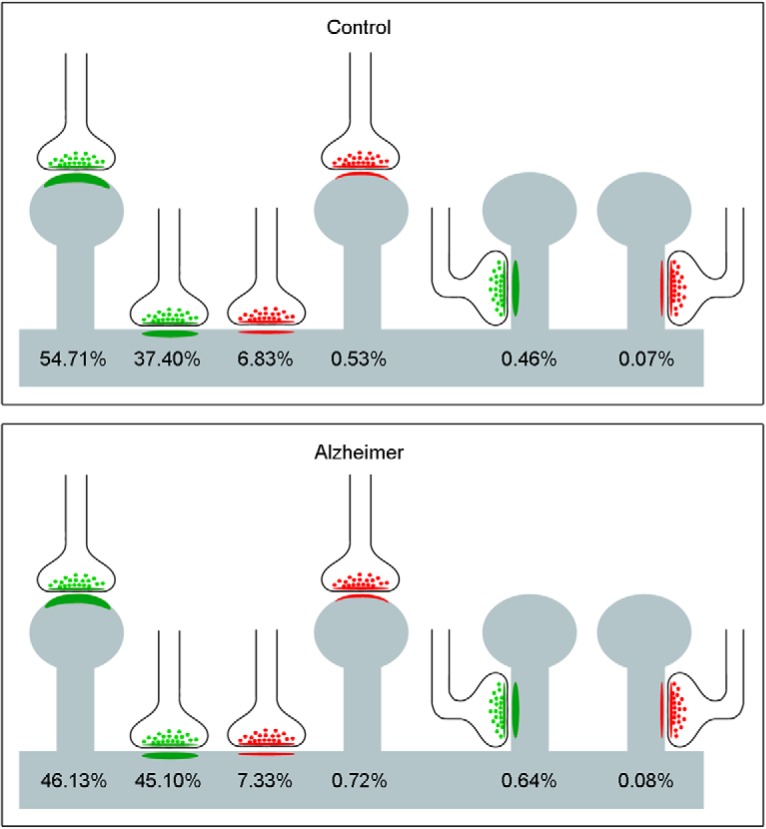
Representation of the distribution of AS (green) and SS (red) on spines and dendritic shafts. Percentages of each type are indicated. Synapses on spines have been sub-classified into those that are established on the head of the spine and those that are established on the neck. AS have been represented in green and SS in red. Control cases are represented on the top and AD patients on the bottom.

When we differentiated the synapses according to their type (AS or SS) and their postsynaptic target (spine heads, spine necks or dendritic shafts), we found that 54.7% were AS targeting spine heads, 37.4% were AS on dendritic shafts, 6.8% corresponded to SS on dendritic shafts, and 0.5% were SS on spine heads (Fig. 4). However, few synapses targeted spine necks (0.5% of AS and 0.1% of SS; Fig. 4).

**Figure 5.**
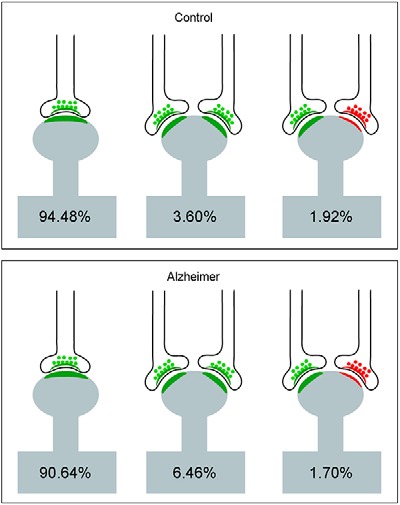
Schematic representation of single and multiple synapses on dendritic spine heads. Percentages of each type are indicated. Synapses on the necks and other combinations that were rarely found (<1%) have not been represented. AS have been represented in green and SS in red. Control cases are represented on the top and AD patients on the bottom.

We also determined the proportion of single or multiple synapses per spine head and found that the majority of synapses were single AS (94.5%). The remaining 5.5% were multiple synapses, which were found on the spine heads in different combinations as follows: 3.6% comprised two AS, with 1.9% comprising one AS and one SS (Fig. 5).

**Figure 6.**
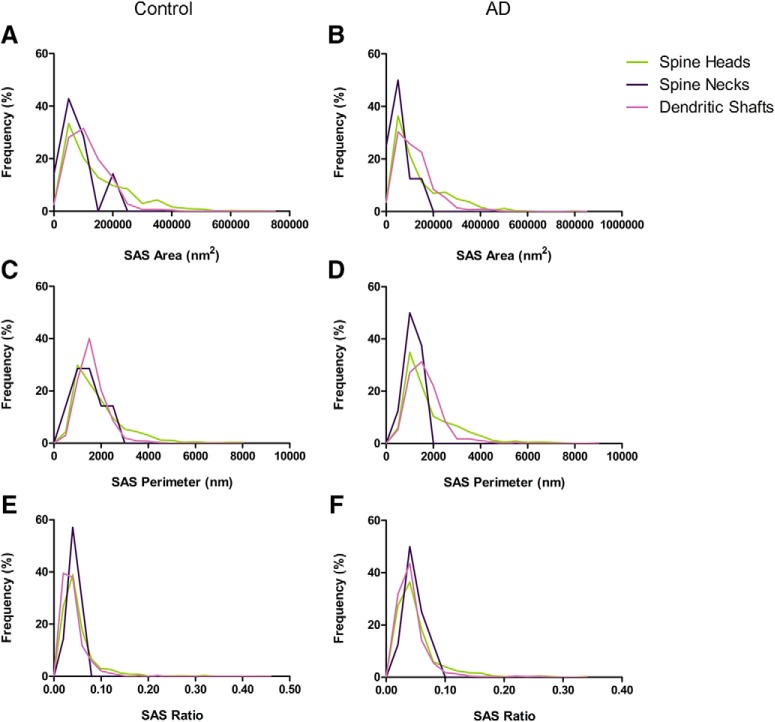
Frequency histograms of the SAS area (***A***, ***B***), perimeter (***C***, ***D***), and curvature (***E***, ***F***) of AS targeting spine heads, spine necks, and dendritic shafts from control cases (***A***, ***C***, ***E***) and from AD patients (***B***, ***D***, ***F***). The mean SAS curvature ratio of synapses targeting spine heads was significantly higher than in synapses targeting dendritic shafts (KW, *p* < 0.0001), both in control cases and AD patients.

Furthermore, we examined the possible relationship between the postsynaptic target of the synapses and their synaptic size. This study was performed by examining the area, perimeter and curvature of the SAS from each AS and SS. In the case of the AS, the mean SAS curvature of synapses targeting spine heads was significantly higher than in synapses targeting dendritic shafts (KW, *p* < 0.001; Table 3; Fig. 6). In the case of SS, the number of synapses was considered too low to perform a robust statistical analysis.

**Table 3.** Data regarding area (nm^2^), perimeter (nm), and curvature (ratio) of the SAS from synapses on spines and dendritic shafts in control cases and AD patients

Group	Postsynaptic structure	Type of synapse	Area of SAS(nm^2^; mean ± SEM)	Perimeter of SAS (nm; mean ± SEM)	Curvature of SAS (mean ± SEM)
Control	Spine heads	AS	14,5013 ± 4103	1877 ± 38	0.05 ± 0.001
		SS	81,295 ± 12,011	1380 ± 138	0.05 ± 0.010
	Spine necks	AS	82,219 ± 23,461	1413 ± 255	0.04 ± 0.004
		SS	19,199 ± 0	627.7 ± 0	0.04 ± 0
	Dendritic shafts	AS	115,703 ± 2845	1613 ± 24	0.04 ± 0.001
		SS	76,129 ± 4299	1442 ± 51	0.06 ± 0.005
Alzheimer	Spine heads	AS	135,310 ± 4762	1815 ± 47	0.05 ± 0.002
		SS	50,292 ± 12,137	1014 ± 128	0.06 ± 0.010
	Spine necks	AS	61,658 ± 13,211	1150 ± 119	0.05 ± 0.005
		SS	55,339 ± 0	1269 ± 0	0.11 ± 0
	Dendritic shafts	AS	119,272 ± 3335	1638 ± 32	0.04 ± 0.001
		SS	78,687 ± 5885	1280 ± 50	0.06 ± 0.005

All data are corrected for shrinkage factor. SEM: standard error of the mean.

#### AD

We examined the postsynaptic targets of 1153 AS and 102 SS that were unambiguously identified. The study of the preferred postsynaptic target revealed that 50.2% of AS were established on spine heads and 0.7% on spine necks. The remaining AS (49.1%) were established on dendritic shafts: 28.0% on aspiny dendritic shafts and 21.1% on spiny shafts (Table 2; Extended Data Table 2-1). In these AD samples, SS also showed a clearly different preference pattern: 90.2% of SS targeted dendritic shafts (49.0% on spiny dendritic shafts and 41.2% on aspiny shafts). A small percentage of SS were established on spine heads (8.8%) and spine necks (1.0%; Table 2; Extended Data Table 2-1).

To evaluate possible preference of AS and SS for spines or dendritic shafts in AD samples, the same analysis performed in control cases was used: χ^2^ test in 2 × 4 and 2 × 2 contingency tables. We found that AS had a significant preference for spine heads: 98.5% of AS versus only 1.5% of SS (χ^2^, *p* < 0.0001). By contrast, the SS showed a significant preference for dendritic shafts (χ^2^, *p* < 0.0001), with 82.9% of AS and 17.1% of SS on dendritic spiny shafts, and 88.5% of AS and 11.5% of SS on aspiny dendritic shafts.

Analysis of synapses according to their type (AS or SS) and their postsynaptic target (spine heads, spine necks and dendritic shafts) showed that, in AD samples, 46.1% of the synapses were AS targeting spine heads, 45.1% were AS on dendritic shafts, 7.3% corresponded to SS located on dendritic shafts and 0.7% to SS on spines (Fig. 4). Similar to observations with the control cases, there were few synapses targeting spine necks (0.7% for AS and 0.1% for SS; Fig. 4).

The presence of single or multiple synapses per spine head was also examined. The majority of synapses established on spine heads were single AS (90.6%) and the least frequent type was single SS (0.2%) on a spine head. The remaining 9.2% were multiple synapses, which were found on the spine heads in different combinations, as follows (Fig. 5): 6.5% comprised two AS, with 1.7% comprising one AS and one SS, and 1.0% comprising two AS and one SS (not shown).

In AD samples, we also evaluated whether the postsynaptic target was related to the size of the synaptic junctions. For this purpose, we examined the area, perimeter and curvature ratio of the SAS from each AS and SS. In the case of the AS, the mean SAS curvature ratio of synapses targeting spine heads was significantly higher than that found in synapses targeting dendritic shafts (KW, *p* < 0.001; Table 3; Fig. 6). In the case of SS, the number of these synapses was not sufficient to perform a robust statistical analysis.

The analysis of SAS features to compare between control and AD samples showed no differences between groups (MW, *p* > 0.05). Likewise, frequency distribution analysis of SAS did not reveal significant differences (KS, *p* > 0.01; Fig. 6).

**Figure 7.**
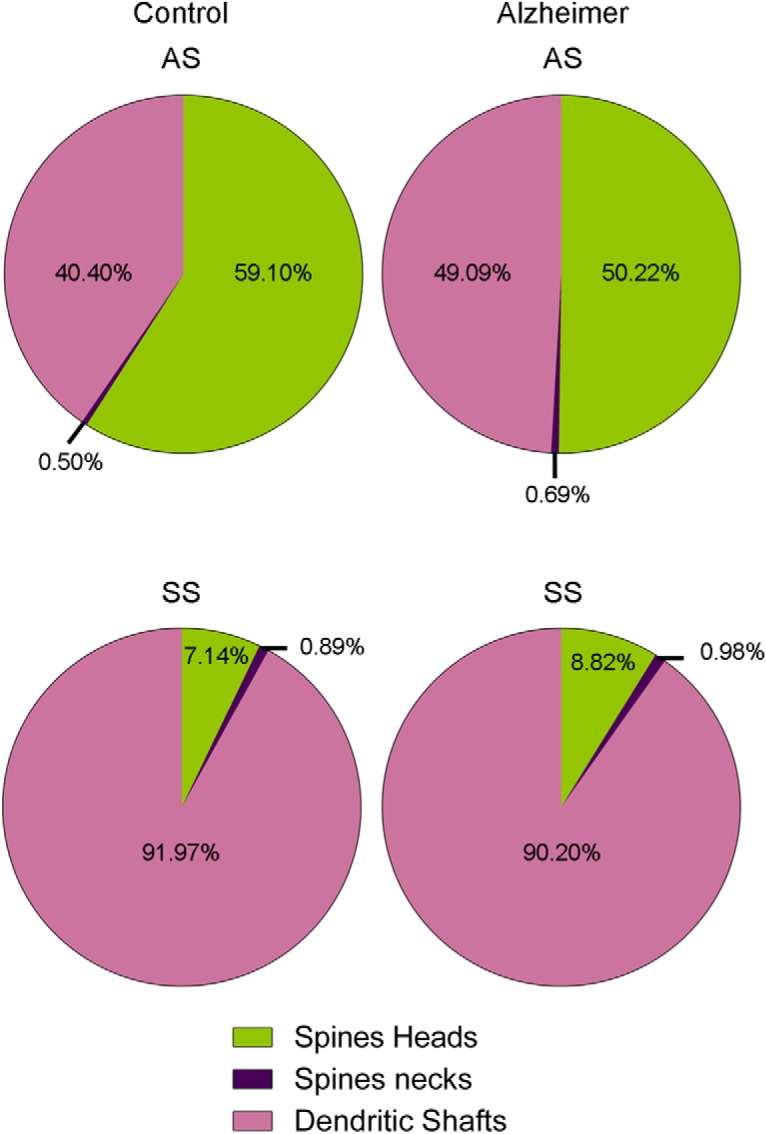
Proportion of AS and SS on spines and dendritic shafts in control and AD cases. In AD patients, there is a significantly lower number of AS targeting spine heads (χ^2^, *p* < 0.001) and a significantly higher number of AS targeting dendritic shafts (χ^2^, *p* < 0.001). Data from SS shows no apparent differences between groups.

To evaluate possible differences in the postsynaptic targets in AD patients compared to control cases, χ^2^ tests in 2 × 4 and 2 × 2 contingency tables were performed. In this case, contingency tables considered both control and AD samples against the type of postsynaptic target. For AS, in AD patients there were significantly less synapses targeting spine heads compared to control cases (χ^2^, *p* < 0.001; Fig. 7). By contrast, we observed that significantly more synapses targeted dendritic shafts in AD patients (*χ^2^*, *p* < 0.001; Fig. 7). No differences were found regarding synapses targeting spine necks (χ^2^, *p =* 0.7099; Fig. 7). Concerning SS, the number of synapses examined was not sufficient to perform a robust statistical analysis.

### The shape and size of the synaptic junctions

Regarding the synaptic shape, synapses were categorized into four main types according to Santuy et al. (2018a): macular, perforated, horseshoe-shaped, and fragmented synapses. Briefly, macular synapses presented continuous disk-shaped PSD; those with holes in the PSDs were classified as perforated synapses; synapses with tortuous horseshoe-shaped perimeters with an indentation were called “horseshoe-shaped”; and irregular small disk-shaped PSDs with no connection between them were considered as “fragmented” synapses (Fig. 1*A*).

#### Control

In control cases, 2561 AS were identified and fully reconstructed in 3D. The majority of these were macular (83.8%), followed by perforated synapses (11.8%). Relatively few were horseshoe-shaped (3.8%), and much fewer still were fragmented synapses (0.6%; Table 4; Extended Data Table 4-1). Regarding SS, we identified and fully reconstructed in 3D a total of 127 synapses. The majority were macular-shaped (83.5%), followed by horseshoe-shaped (12.6%). The least frequent type was the perforated (3.9%) and no fragmented SS were found (Table 4; Extended Data Table 4-1).

**Table 4.** Proportion of the different shapes of synaptic junctions in control and AD patients

Group	Type of synapse	Macular synapses	Perforated synapses	Horseshoe-shaped synapses	Fragmented synapses	Total synapses
Control	AS	83.8% (2145)	11.8% (302)	3.8% (98)	0.6% (16)	100% (2561)
	SS	83.5% (106)	3.9% (5)	12.6% (16)	0.0% (0)	100% (127)
Alzheimer	AS	81.4% (1558)	11.5% (221)	5.3% (101)	1.8% (35)	100% (1915)
	SS	92.5% (110)	6.7% (8)	0.8% (1)	0.0% (0)	100% (119)

Data are given as percentages with the absolute number of synapses studied in parentheses. Data for each individual case are represented in Table 4-1.

10.1523/ENEURO.0140-19.2019.t4-1 Extended Data Table 4-1 Proportion of the different shapes of synaptic junctions for each case Download Table 4-1, DOCX file.

To evaluate possible association of AS and SS regarding the synaptic shape, again χ^2^ tests in 2 × 4 and 2 × 2 contingency tables were created, considering both types of synapses against the morphological synaptic type. The null hypotheses of these χ^2^ tests were “H_0_: type of synapse and morphological synaptic type are independent.” We found that 95.3% of macular synapses were AS and 4.7% were SS. This proportion was slightly different in perforated synapses (98.4% AS and 1.6% SS), but in the case of horseshoe-shaped synapses, the proportion of AS was significantly lower (86.0% were AS and 14% SS; χ^2^, *p* < 0.0001), indicating that the horseshoe-shaped synapses were more frequent among SS than AS. Regarding the fragmented synapses, 100% were AS (Fig. 1*C*).

**Figure 8.**
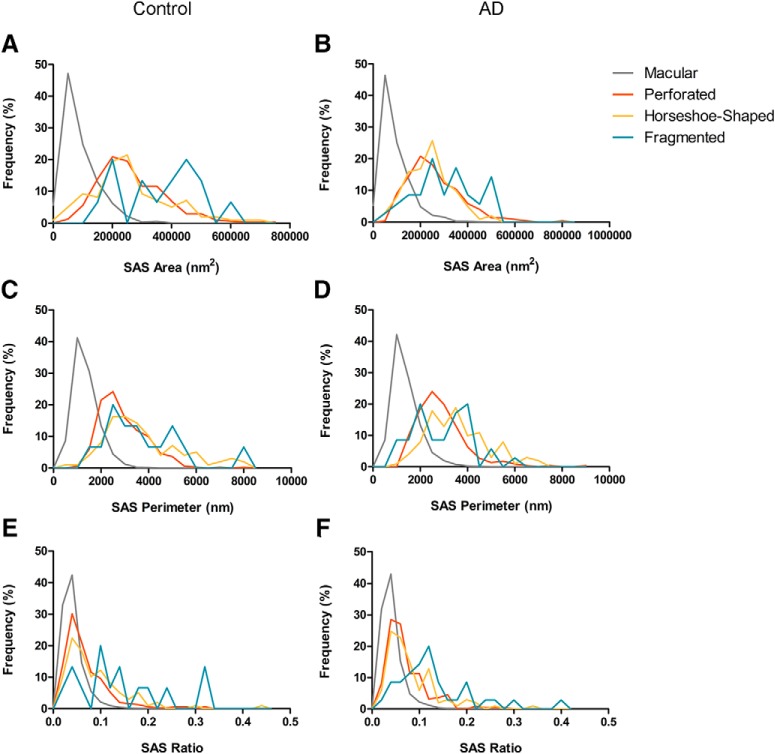
Frequency histograms of the SAS area (***A***, ***B***), perimeter (***C***, ***D***), and curvature (***E***, ***F***) of macular, perforated, horseshoe-shaped and segmented AS from control cases (***A***, ***C***, ***E***) and from AD patients (***B***, ***D***, ***F***). The mean SAS area, perimeter and curvature of macular synapses was significantly smaller than in perforated, horseshoe-shaped and segmented synapses (KW, *p* < 0.0001), both in control cases and AD patients.

We also determined whether the shape of the synapses was associated with differences in their size. For this purpose, we examined the area, perimeter and curvature ratio of the SAS from each AS and SS. In the case of the AS, the mean SAS area, perimeter and curvature ratio of macular synapses were all significantly smaller than in perforated, horseshoe-shaped and fragmented synapses (KW, *p* < 0.0001; Table 5; Fig. 8). Although we observed the same tendency in the case of SS (smaller macular synapses than perforated and horseshoe-shaped synapses), only five perforated synapses and 16 horseshoe-shaped ones were found, and therefore, statistical analysis was not applied.

**Table 5.** Data regarding area (nm^2^), perimeter (nm), and curvature (ratio) of the SAS of macular, perforated, horseshoe-shaped, and fragmented synapses in control cases and AD patients

Group	Shape of synapses	Type of synapse	Area of SAS (nm^2^; mean ± SEM)	Perimeter of SAS (nm; mean ± SEM)	Curvature of SAS (mean ± SEM)
Control	Macular	AS	88,272 ± 1283	1352 ± 11	0.04 ± 0.001
		SS	70,960 ± 3764	1306 ± 40	0.05 ± 0.005
	Perforated	AS	264,960 ± 6606	2914 ± 58	0.07 ± 0.003
		SS	108,320 ± 22,066	2080 ± 279	0.07 ± 0.016
	Horseshoe-shaped	AS	262,251 ± 13,757	3725 ± 163	0.09 ± 0.007
		SS	88,466 ± 13204	1876 ± 153	0.06 ± 0.011
	Fragmented	AS	360,245 ± 34,068	3735 ± 435	0.14 ± 0.025
		SS	-	-	-
Alzheimer	Macular	AS	91,162 ± 1689	1367 ± 14	0.04 ± 0.001
		SS	59,436 ± 3156	1224 ± 42	0.06 ± 0.005
	Perforated	AS	259,540 ± 7942	2879 ± 73	0.07 ± 0.003
		SS	123,147 ± 14,857	1776 ± 129	0.03 ± 0.006
	Horseshoe-shaped	AS	237,778 ± 9524	3568 ± 127	0.08 ± 0.006
		SS	115,312 ± 0.0	2431 ± 0	0.07 ± 0.000
	Fragmented	AS	303,922 ± 21,783	2985 ± 210	0.13 ± 0.014
		SS	-	-	-

All data are corrected for shrinkage factor. SEM: standard error of the mean.

#### AD

In AD patients, a total of 1915 AS were identified and fully 3D reconstructed. The majority of these synapses were macular-shaped (81.4%), followed by perforated synapses (11.5%), horseshoe-shaped (5.3%), and only 1.8% were fragmented synapses. Concerning SS, we identified and fully 3D reconstructed a total of 119 synapses. The majority were macular (92.5%), followed by perforated (6.7%). The least frequent type was the horseshoe-shaped (0.8%), and similar to the observations with control cases, fragmented SS were not found in AD patients (Table 4; Extended Data Table 4-1).

As occurred in the control cases, we found that 93.4% of macular synapses were AS and 6.6% were SS. This proportion was slightly different in perforated synapses (96.5% AS and 3.5% SS) and horseshoe-shaped synapses (99.0% AS and 1.0% SS). Regarding the fragmented synapses, 100% were AS (Fig. 1*D*).

To evaluate possible differences in the proportion of the synaptic shapes between control cases and AD patients, χ^2^ tests in 2 × 4 and 2 × 2 contingency tables were performed. In this case, contingency tables considered both control and AD samples against the morphological synaptic type. For AS the results indicated that, in AD patients, fragmented synapses were more frequent than in control cases (χ^2^, *p* < 0.001). This difference was not significant regarding macular, perforated and horseshoe synapses (χ^2^, *p* > 0.01; Fig. 1*B*), but we observed a slightly higher number in horseshoe synapses (χ^2^, *p* = 0.024) and slightly fewer number in macular synapses (χ^2^, *p* = 0.039). Regarding SS, the number of these synapses was not sufficient to perform a robust statistical analysis.

Moreover, we determined whether the shape of the synapses was associated with differences in their size. The area, perimeter and curvature of the SAS from each of the AS and SS were examined. In the case of the AS, the mean SAS area, perimeter and curvature of macular synapses were, as occurred in control cases, significantly smaller than in perforated, horseshoe-shaped and fragmented synapses (KW, *p* < 0.0001; Table 5; Fig. 8). Although we observed the same tendency in the case of SS (smaller macular synapses than perforated and horseshoe-shaped synapses), the number of these synapses was not sufficient to perform a robust statistical analysis. SAS features of AS such as the area, perimeter and the curvature were compared between the two groups, with no significant differences observed between control cases and AD patients (MW, *p* > 0.05). Likewise, frequency distribution analysis did not reveal significant differences (KS, *p* > 0.01).

## Discussion

This study provides the following findings: first, both in control cases and AD patients, AS had a significant preference for spine heads, while the preference of SS was for dendritic shafts. However, in AD patients we observed a lower percentage of synapses targeting spine heads. Second, regarding the shape of synapses, in control and AD samples, the vast majority of synapses had a macular shape (83.8% for AS and 83.5% for SS control; 81.4% for AS and 92.5% for SS AD). Third, in AD patients, we observed a higher percentage of fragmented AS than in control cases.

### Distribution of postsynaptic targets

#### Synaptic targets in control cases

The analysis of the preferred postsynaptic target revealed that the vast majority of AS were established on spines, whereas, in the case of SS, the vast majority were on dendritic shafts. If we take into account the synaptic type (AS or SS) and its postsynaptic target, we found that in control cases 54.7% of synapses were AS targeting spine heads, 37.4% were AS on dendritic shafts, 6.8% were SS located on dendritic shafts, and 0.5% were SS on spine heads. Very few synapses targeted spine necks (0.5% of AS and 0.1% of SS).

Since there are no similar studies performed in the human cerebral cortex, we compared our results with EM studies performed in the neuropil of other species and cortical regions (Table 6; Beaulieu and Colonnier, 1985; Beaulieu et al., 1992; Micheva and Beaulieu, 1996). These studies include data from the monkey striate cortex, the cat visual cortex, and the rat barrel cortex. As summarized in Table 6, there is a general pattern in all cortical areas and species: AS located on spines predominate, followed by AS located on dendritic shafts, SS located on dendritic shafts, and, finally, SS located on spines. Taking into account the considerable differences in the functional and structural features between the different cortical areas and species (DeFelipe, 2011), the similarity of this general pattern of synaptic distribution is remarkable. Nevertheless, there are important differences in the percentage for each category, which could be attributed to species specialization of the different cortical areas examined. For example, Layer II of the human TEC had the lowest percentage of AS on spines, although this percentage was similar to that found in layer 2/3 of the monkey striate cortex. However, the proportions of AS on shafts and SS on spines were largest and lowest, respectively, in the human TEC (Table 6). Nevertheless, we cannot rule out the possibility that the differences in the percentages observed in the present study and the above mentioned publications in experimental animals could also be explained, at least in part, by the different methodological approaches used to identify the postsynaptic targets; we used the gold standard method, namely full reconstructions of the synapses, rather than the partial reconstructions conducted in other publications.

**Table 6.** Summary of the data from the studies of the pattern of synaptic distribution

	Human TEC	Striate cortex (area 17) of the monkey^1^		Visual cortex of the adult cat^2^		Somatosensory cortex of the adult rat (P60)^3^
						
	Layer II	Layer II−III	All layers	Layer II	All layers	All layers
AS on spines	55.2%	54.9%	53.0%	71.2%	66.4%	73.0%
AS on dendritic shafts	37.4%	27.7%	30.0%	13.3%	17.6%	12.9%
SS on dendritic shafts	6.7%	11.4%	11.9%	10.8%	10.6%	9.3%
SS on spines	0.61%	6.0%	5.1%	4.7%	5.3%	4.8%

Data are given as percentages. Data were taken from: ^1^Beaulieu et al., 1992; ^2^Beaulieu and Colonnier (1985); ^3^Micheva and Beaulieu (1996).

#### Synaptic targets in AD

In the AD samples, the study of the preferred postsynaptic target showed that, as observed with control cases, the majority of AS were established on spines (with 50.2% targeting spine heads and 0.7% targeting spine necks). The remaining AS were established on dendritic shafts (28.0% of AS on aspiny dendritic shafts and 21.1% of AS on spiny shafts). However, the majority of SS target dendritic shafts (49.0% on spiny dendritic shafts and 41.2% on aspiny shafts), and a small percentage of SS were established on spine heads (8.8%) and spine necks (1.0%).

Comparison of the postsynaptic targets in AD patients and control cases revealed a significant reduction in the number of synapses targeting spine heads in AD patients. This reduction is probably related to the changes in number and morphology of spines that occur in AD. For example, in the vicinity of amyloid-plaques in the cerebral cortex of mouse models of AD, as well as in tissue from AD patients, spines are loss or undergo a variety of morphological changes (Spires-Jones et al., 2007; Knafo et al., 2009; Tackenberg et al., 2009; Merino-Serrais et al., 2011; Pozueta et al., 2013; Llorens-Martín et al., 2014; Zou et al., 2015). Furthermore, some studies have observed loss and morphological changes of spines in tau transgenic mice and AD patients, related to the presence of pretangles or neurofibrillary tangles in pyramidal neurons (Tackenberg et al., 2009; Merino-Serrais et al., 2013; Pozueta et al., 2013). Thus, both Aβ-plaques in the neuropil and the intracellular neurofibrillary tangles within pyramidal cells induce alterations in spines. Since the majority of the synapses a pyramidal cell receives are on spines and they represent the vast majority of AS synapses (DeFelipe and Fariñas, 1992), the loss of spines, and, therefore, the loss of synapses, has been proposed as the structural basis of pathogenesis in AD and in other neurodegenerative diseases, such as Huntington’s disease, and amyotrophic lateral sclerosis-associated dementia (Herms and Dorostkar, 2016).

Despite the fact that we found a significantly lower number of synapses targeting spine heads in AD patients, it is important to note that there was a high interindividual variability (Extended Data Table 2-1). Two AD patients (IF6, VK11), who had the lowest Braak/CERAD stage (IIIIV/A), showed values of synapses targeting spine heads similar to control cases. By contrast, one AD patient (VK22) classified with the highest Braak/CERAD stage (V/C) presented the lowest number of synapses targeting spine heads. Therefore, the number of synapses targeting spine heads might be modified by differences in the disease progression. By contrast, one AD patient (IF1), classified as Braak/CERAD stage IV/B, showed values of synapses targeting spine heads similar to control cases. Since this patient apparently did not display cognitive impairment, it is possible that this particular case may represent a predementia stage of AD with no impairment of spines. This variability could not be explained by technical effects, since postmortem delays were all similar and we used the same methodological procedures. However, others factors such as gender or aging could explain the variability among cases, since these factors may influence the number of synapses (Alonso-Nanclares et al., 2008; Peters and Kemper, 2012).

### The shape and size of the synaptic junctions: controls versus AD

It is not only the number of spines that is essential in terms of maintenance of synaptic connectivity and plasticity; spine morphology is also critical from a functional point of view (Yuste, 2010; Herms and Dorostkar, 2016). The spine head volume has been related to the area of the PSD, which has in turn been related to the number of postsynaptic receptors (Nusser et al., 1998) and the probability of neurotransmitter release (Arellano et al., 2007; Montes et al., 2015). Regarding the molecular composition of the PSD, the larger the synapse, the higher the actual number of AMPA receptors, whereas a higher concentration of NMDA receptors has been found in smaller synapses (Kharazia and Weinberg, 1999). In the present study, no changes in any of the PSD size parameters (SAS area and perimeter) were found in AD samples. However, the protein composition of human synapses is highly complex (Grant, 2012) and we cannot rule out changes in the composition and number of postsynaptic receptors and other elements of the postsynaptic signaling complexes in Layer II of the TEC in AD patients. Further studies of the human synapse proteome in the TEC would be necessary to better understand the synaptic changes found in this cortical region.

In both control and AD cases, synaptic junctions were categorized into four main types depending on their shape: macular, perforated, horseshoe-shaped and fragmented synapses.

#### Control

In general, the vast majority of both AS and SS had a macular shape; followed far behind, the second most common type was perforated synapses in the case of AS, or horseshoe-shaped synapses in the case of SS. Fragmented synapses were found in low numbers and all of them were AS. Regarding the average size of synapses, macular synapses were smaller than perforated, horseshoe-shaped and fragmented synapses. Unfortunately, since there are no studies about the 3D synaptic morphology in the human neocortex, our results cannot be compared with previous studies performed in the TEC or in any other human cortical region. These results are similar to previous studies on synaptic shape and size performed in the juvenile rat somatosensory cortex (Santuy et al., 2018a).

Although we do not know how morphological synaptic changes affect synaptic function, some studies have related them to an increase in the synaptic transmission efficiency, because the remodeling process involves the insertion of new receptors in the postsynaptic membrane (Lüscher et al., 2000). Moreover, in response to synaptic activity, receptors can be incorporated into the PSD either by endosomal pathways or by lateral diffusion from the extrasynaptic membrane zone (Kneussel and Hausrat, 2016). Under normal conditions, the macular synapses had the capacity to become larger by progressively adopting a more tortuous perimeter with perforations and indentations (Geinisman et al., 1987, 1991, 1992a,b). So, synapses may become larger and more complex by a remodeling process involving the incorporation of receptors into the postsynaptic membrane. Accordingly, some studies have reported higher immunoreactivity for glutamate receptors (AMPA and NMDA) in perforated synapses than in non-perforated ones (Ganeshina et al., 2004a,b). Models to study how variations in size of synaptic junctions are related to characteristics such as release probability of neurotransmitter and density of postsynaptic AMPA receptors suggest that large synapses with more number of postsynaptic receptors would produce stronger and more homogeneous responses, while small synapses with fewer receptors would produce weaker and much more variable responses (Montes et al., 2015).

Furthermore, it has been postulated that fragmented synapses are specialized synapses which would lead to a greater synaptic response than in the case of synapses that only have a single PSD (Geinisman et al., 1987; Ganeshina et al., 2004a). Several studies have found an increase in the number of fragmented synapses resulting from the turnover of original synapses in the dental gyrus and CA1 of the rat hippocampus after LTP or kindling stimulation (Geinisman et al., 1991, 1992a,b, 1993; Toni et al., 2001). In this process, a continuous disk-shaped PSD synapse (macular) enlarges with the formation of one or more holes in its PSD, becoming a horseshoe shape before finally separating into different PSDs with no connections between them (Geinisman et al., 1987). Thus, fragmented synapses could be structural intermediates in various forms of synaptic dynamics and their increase in their number could be responsible for the enhancement of synaptic efficacy that is typical of LTP and kindling. Finally, if perforated, horseshoe-shaped and fragmented synapses represent different dynamic functional stages, and the percentage of these synapses is relatively low, it follows that dynamic synaptic changes must be of relatively little importance.

However, we should keep in mind that, in the TEC, there are billions of synapses (510 × 10^6^ synapses per mm^3^; Domínguez-Álvaro et al., 2018). Thus, there are millions of synapses with these different shapes, which could in fact be interpreted as a relatively high rate of synaptic dynamism.

#### AD

In AD cases, the vast majority of both AS and SS were macular, followed by perforated, horseshoe-shaped and fragmented synapses. In the case of SS, the percentages of perforated and horseshoe-shaped synapses were lower than for AS, and fragmented synapses were not found.

When we compared the proportion of the different synaptic shapes between control cases and AD patients, we found that AS fragmented synapses were more frequent in AD patients than in control cases. This difference was not found in macular, perforated or horseshoe-shaped synapses, although a slightly lower proportion of macular synapses and a slightly higher proportion of horseshoe synapses were found in AD patients.

In our AD samples, we also determined whether the shape of the synapses was related to the synaptic size (area, perimeter and curvature of the SAS). We observed that synapses in AD samples had similar morphological features to those observed in control cases. In the case of the AS, the area, perimeter and curvature of macular synapses were significantly smaller than in perforated, horseshoe-shaped, and fragmented synapses. Moreover, regarding these synaptic features, there were no significant differences between control and AD patients, as previously reported in other cortical regions using conventional transmission EM (Scheff and Price, 2006; Scheff et al., 1993).

Since an increase in the number of fragmented synapses has been related to potentiation of the synaptic efficacy (Geinisman et al., 1991, 1992a,b, 1993; Toni et al., 2001), the increased number of this type of synapse found in AD patients could be due to the activation of compensatory mechanisms in response to the synaptic loss that occurs in this cortical region (Domínguez-Álvaro et al., 2018). Accordingly, a higher number of perforated synapses have been reported in several diseases and under certain experimental conditions, such as, e.g., after striatal dopamine depletion in the striatum of the rat (Anaya-Martínez et al., 2014). Recently, enhancement of physiologic synaptic activity during early stages of AD has been suggested as a compensatory response (Tampellini, 2015). Since the potentiation of synaptic activity may promote an increase in the proportion of fragmented synapses, we could hypothesize that the higher proportion of fragmented synapses found in AD patients could be a compensatory mechanism during the progression of this disease, which would end in the generation of more fragmented synapses. However, it could be possible that this kind of mechanism fails and synaptic activity would fall off. Moreover, as fragmented synapses have more than one independent release site their formation could involve an increase in glutamate release, which may promote excitotoxicity and neuronal death (Anaya-Martínez et al., 2014).

Finally, although we analyzed only five control cases and five AD patients, it is important to keep in mind that, thanks to FIB/SEM technology, it was possible to obtain the first largest collection of 3D reconstructed synapses of the human brain, with thousands of synapses examined. This allowed us to accurately determine their morphology and postsynaptic targets, constituting firm support for the evidence provided by the data presented. Nevertheless, further verification involving the examination of more cases with similar ages would be necessary to better understand the possible differences in the synaptic organization between controls and AD. As a final point, it is well established that there are differences in the microstructure of the cerebral cortex depending on the cortical area and layer (DeFelipe, 2011). Therefore, the data obtained in the present study probably cannot be extrapolated to other cortical regions. In other words, the synaptic organization must be examined separately in each particular region and for different ages and genders. This will make it possible to determine the similarities and differences in the synaptic organization between different cortical regions of the human brain.

### Concluding remarks

In summary, this study provides evidence of a reduction in the number of synapses targeting spine heads in AD patients, in line with previous studies. The loss of spines, and, therefore, the loss of synapses, has been proposed as the structural basis of pathogenesis in AD. Besides, in this study we observed morphological synaptic alterations in TEC in AD patients. How these morphological changes could affect the synaptic function is unknown, but these changes have been related to modifications in the synaptic transmission efficiency. Thus, the increase in the number of fragmented synapses in AD patients could be due to the activation of compensatory mechanisms in response to the synaptic loss that occurs in this cortical region during the progression of the disease. However, it could be possible that this kind of mechanism fails and synaptic activity would fall off.

## Synthesis

Reviewing Editor: Alfonso Araque, University of Minnesota Decisions are customarily a result of the Reviewing Editor and the peer reviewers coming together and discussing their recommendations until a consensus is reached. When revisions are invited, a fact-based synthesis statement explaining their decision and outlining what is needed to prepare a revision will be listed below. The following reviewer(s) agreed to reveal their identity: George Perry, James C. Vickers.

The reviewers have found high merit and interest on the findings reported. I concur with the reviewers' comments expressing the high interest of the study. However, they also expressed some concerns about several issues that need to be addressed and clarified. I also consider pertinent the concerns expressed. I believe that addressing those concerns, which will definitely improve the manuscript, is necessarily required to firmly validate the results and support the conclusions.

Specific comments of the reviewers:

Reviewer 1.

A wonderful study, It clarifies that synapse changes in AD are subtle, much like Scheff but much higher quality. The major weakness I see is use of autopsy tissue. This study could have been done with biopsy tissue. This point does not mean all should be redone---but it should be discussed. The should should make it clear that the synapse changes are small, it is not as often stated that everything is dead. The synapse changes cannot explain why the clinical trials have failed. This should be clearly stated.

1. Title should mention it is a 3D study--it is head and shoulders over prior work.

2.The work could gain from considering the structural and physiology studies of N Robbins on the neuromuscular junction. The findings are complementary.

3. Diagnostic criteria need to be exactly specified.

4. Statistics review is essential. It requires professional stat analysis.

Reviewer 2.

It is a great pleasure to see the fine detail of the human brain - the quality of the ultrastructural detail of the data is a clear strength of this paper.

The n is small for both control and AD case types. This is understandable given the vast amount of data that can be derived from FIB SEM approaches. The quality of the ultrastructure obtained in outstanding for human brain preparations for ultrastructural analysis. It would be useful to know whether all samples provided high quality data? Were there any factors that affected such analysis such as agonal state or post-mortem preparation?

The non-AD cases are years of age and therefore may be expected to have some degree of neurofibrillary pathology in layer II of this entorhinal region. Were these encountered by the FIB SEM analysis? These would likely be intracellular tangles which could affect the morphology of processes and synaptic innervation. There would be many tangles in this region in the AD cases - were these identified?

The AD cases would be expected to contain beta amyloid plaques, but this does not seem to be mentioned. This may be important as other immunohistochemical studies have shown that were dense plaques occur, there is obliteration of synapses, and there is spine loss in surrounding dendrites. Some of the control cases may also contain a degree of amyloid deposition. It may be that there was very little amyloid deposition in layer II of this entorhinal region.

There have also been previous studies on the phenotyping of synapse loss based on immunohistochemical markers that could be useful in the context of changes synapses described in the current paper, particularly in regard to excitatory and inhibitory synpases.

I understand that the analytical focus has been on proportions of synapse types and their morphology, but is it possible to extract an estimation of overt potential loss of synapses in these sets of images from AD cases? In neocortex, it has been estimated that between 30-40% of synapses are lost based on immunohistochemical methods.

Are there quality control procedures to check whether the EspINA software is accurately delineating different synapse types?
