# Supplementary material for: 3D Electron Microscopy Study of Synaptic Organization of the Normal Human Transentorhinal Cortex and Its Possible Alterations in Alzheimer’s Disease
Source: eNeuro. 2019 Jul 9;6(4):ENEURO.0140-19.2019. doi: 10.1523/ENEURO.0140-19.2019 (PMC6620390; doi:10.1523/ENEURO.0140-19.2019)
Supplement: Extended Data Table 2-1 — Distribution of AS and SS on spines and dendritic shafts for each individual case Download Table 2-1, DOCX file. [file sup_enu-eN-NWR-0140-19-s03.docx]

**Table 2-1. Distribution of asymmetric (AS) and symmetric (SS) synapses on spines and dendritic shafts for each individual case**

| **Case** | **Type of synapse** | **Synapses on spine heads** | **Synapses on spine necks** | **Synapses on aspiny dendritic shaft** | **Synapses on spiny dendritic shaft** | **Total No. of synapses** |
| --- | --- | --- | --- | --- | --- | --- |
| AB1 | AS | 60.7% (153) | 0.8% (2) | 22.2% (56) | 16.3% (41) | 100% (252) |
|  | SS | 13.6% (3) | 0% (0) | 36.4% (8) | 50.0% (11) | 100% (22) |
| AB2 | AS | 53.2% (91) | 0.6% (1) | 25.7% (44) | 20.5% (35) | 100% (171) |
|  | SS | 0% (0) | 6.3% (1) | 62.5% (10) | 31.2% (5) | 100% (16) |
| IF10 | AS | 48.7% (150) | 0.7% (2) | 22.7% (70) | 27.9% (86) | 100% (308) |
|  | SS | 4.0% (1) | 0% (0) | 44.0% (11) | 52.0% (13) | 100% (25) |
| M16 | AS | 58.6% (238) | 0.5% (2) | 17.2% (70) | 23.7% (96) | 100% (406) |
|  | SS | 8.3% (2) | 0% (0) | 16.7% (4) | 75.0% (18) | 100% (24) |
| M17 | AS | 74.5% (193) | 0% (0) | 14.7% (38) | 10.8% (28) | 100% (259) |
|  | SS | 8.0% (2) | 0% (0) | 40.0% (10) | 52.0% (13) | 100% (25) |
| IF1 | AS | 59.9% (161) | 0.4% (1) | 16.7% (45) | 23.0% (62) | 100% (269) |
|  | SS | 17.4% (4) | 0% (0) | 26.1% (6) | 56.5% (13) | 100% (23) |
| IF2 | AS | 45.7% (116) | 1.2% (3) | 23.6% (60) | 29.5% (75) | 100% (254) |
|  | SS | 25.0% (5) | 0% (0) | 20.0% (4) | 55.0% (11) | 100% (20) |
| IF6 | AS | 56.8% (151) | 0.4% (1) | 24.4% (65) | 18.4% (49) | 100% (266) |
|  | SS | 0% (0) | 0% (0) | 33.3% (8) | 66.7% (16) | 100% (24) |
| VK11 | AS | 46.9% (127) | 0.4% (1) | 35.4% (96) | 17.3% (47) | 100% (271) |
|  | SS | 0% (0) | 0% (0) | 64.0% (16) | 36.0% (9) | 100% (25) |
| VK22 | AS | 25.8% (24) | 2.2% (2) | 61.3% (57) | 10.7% (10) | 100% (93) |
|  | SS | 0% (0) | 10.0% (1) | 80.0% (8) | 10.0% (1) | 100% (10) |
