# Supplementary material for: 3D Electron Microscopy Study of Synaptic Organization of the Normal Human Transentorhinal Cortex and Its Possible Alterations in Alzheimer’s Disease
Source: eNeuro. 2019 Jul 9;6(4):ENEURO.0140-19.2019. doi: 10.1523/ENEURO.0140-19.2019 (PMC6620390; doi:10.1523/ENEURO.0140-19.2019)
Supplement: Extended Data Table 2-2 — An example of a 2 × 4 contingency table showing the type of synapse against the type of postsynaptic target in control cases Download Table 2-2, DOCX file. [file sup_enu-eN-NWR-0140-19-s04.docx]

**Table 2-2. An example of a 2x4 contingency table showing the type of synapse against the type of postsynaptic target in control cases.**

|  |  | Type of postsynaptic target | | | |  |
| --- | --- | --- | --- | --- | --- | --- |
|  |  | Spine heads | Spine necks | Aspiny dendritic shafts | Spiny dendritic shafts | Totals |
| Type of synapse | AS | **825** | **7** | **278** | **286** | 1396 |
|  | SS | **8** | **1** | **43** | **60** | 112 |
|  | Totals | 833 | 8 | 321 | 346 | 1508 |
