# Supplementary material for: 3D Electron Microscopy Study of Synaptic Organization of the Normal Human Transentorhinal Cortex and Its Possible Alterations in Alzheimer’s Disease
Source: eNeuro. 2019 Jul 9;6(4):ENEURO.0140-19.2019. doi: 10.1523/ENEURO.0140-19.2019 (PMC6620390; doi:10.1523/ENEURO.0140-19.2019)
Supplement: Extended Data Table 2-3 — Three examples of 2 × 2 contingency tables showing the type of synapse against the type of postsynaptic target in control cases Download Table 2-3, DOCX file. [file sup_enu-eN-NWR-0140-19-s05.docx]

**Table 2-3. Three examples of 2x2 contingency tables showing the type of synapse against the type of postsynaptic target in control cases.**

|  |  | Type of postsynaptic target | | |  |
| --- | --- | --- | --- | --- | --- |
|  |  | Spine heads | Non-spine heads | | Totals |
| Type of synapse | AS | **825** (771.13) | **571** (624.87) | | 1396 |
|  | SS | **8** (61.87) | **104** (50.13) | | 112 |
|  | Totals | 833 | 675 | | 1508 |
|  |  | Type of postsynaptic target | | |  |
|  |  | Aspiny dendritic shafts | | Non-aspiny dendritic shafts | Totals |
| Type of synapse | AS | **278** (297.16) | | **1118** (1098.84) | 1396 |
|  | SS | **43** (23.84) | | **69** (88.16) | 112 |
|  | Totals | 321 | | 1187 | 1508 |
|  |  | Type of postsynaptic target | | |  |
|  |  | Spiny dendritic shafts | Non-spiny dendritic shafts | | Totals |
| Type of synapse | AS | **286** (320.3) | **1110** (1075.7) | | 1396 |
|  | SS | **60** (25.7) | **52** (86.3) | | 112 |
|  | Totals | 346 | 1162 | | 1508 |
