# Supplementary material for: 3D Electron Microscopy Study of Synaptic Organization of the Normal Human Transentorhinal Cortex and Its Possible Alterations in Alzheimer’s Disease
Source: eNeuro. 2019 Jul 9;6(4):ENEURO.0140-19.2019. doi: 10.1523/ENEURO.0140-19.2019 (PMC6620390; doi:10.1523/ENEURO.0140-19.2019)
Supplement: Extended Data Table 4-1 — Proportion of the different shapes of synaptic junctions for each case Download Table 4-1, DOCX file. [file sup_enu-eN-NWR-0140-19-s06.docx]

**Table 4-1. Proportion of the different shapes of synaptic junctions for each case**

| **Case** | **Type of synapse** | **Macular synapses** | **Perforated synapses** | **Horseshoe-shaped synapses** | **Fragmented synapses** | **Total No. of synapses** |
| --- | --- | --- | --- | --- | --- | --- |
| **AB1** | AS | 79.3% (318) | 13.9% (56) | 6.5% (26) | 0.3% (1) | 100% (401) |
|  | SS | 84.0% (21) | 0% (0) | 16.0% (4) | 0% (0) | 100% (25) |
| **AB2** | AS | 88.5% (383) | 7.4% (32) | 3.9% (17) | 0.2% (1) | 100% (433) |
|  | SS | 81.3% (13) | 0% (0) | 18.7% (3) | 0% (0) | 100% (16) |
| **IF10** | AS | 84.3% (348) | 13.3% (55) | 2.4% (10) | 0% (0) | 100% (413) |
|  | SS | 84.6% (22) | 0% (0) | 15.4% (4) | 0% (0) | 100% (26) |
| **M16** | AS | 88.8% (524) | 7.8% (46) | 3.1% (18) | 0.3% (2) | 100% (590) |
|  | SS | 85.8% (24) | 7.1% (2) | 7.1% (2) | 0% (0) | 100% (28) |
| **M17** | AS | 79.0% (572) | 15.6% (113) | 3.7% (27) | 1.7% (12) | 100% (724) |
|  | SS | 81.2% (26) | 9.4% (3) | 9.4% (3) | 0% (0) | 100% (32) |
| **IF1** | AS | 86.3% (303) | 6.8% (24) | 4.9% (17) | 2.0% (7) | 100% (351) |
|  | SS | 96.0% (24) | 0% (0) | 4.0% (1) | 0% (0) | 100% (25) |
| **IF2** | AS | 85.0% (379) | 13.0% (58) | 1.1% (5) | 0.9% (4) | 100% (446) |
|  | SS | 100% (25) | 0% (0) | 0% (0) | 0% (0) | 100% (25) |
| **IF6** | AS | 78.3% (342) | 10.5% (46) | 8.9% (39) | 2.3% (10) | 100% (437) |
|  | SS | 79.3% (23) | 20.7% (6) | 0% (0) | 0% (0) | 100% (29) |
| **VK11** | AS | 76.6% (416) | 14.8% (80) | 6.6% (36) | 2.0% (11) | 100% (543) |
|  | SS | 93.1% (27) | 6.9% (2) | 0% (0) | 0% (0) | 100% (29) |
| **VK22** | AS | 85.5% (118) | 9.4% (13) | 2.9% (4) | 2.2% (3) | 100% (138) |
|  | SS | 100% (11) | 0% (0) | 0% (0) | 0% (0) | 100% (11) |
